# Selenium maintains Ca^2+^ homeostasis in sheep lymphocytes challenged by oxidative stress

**DOI:** 10.1371/journal.pone.0201523

**Published:** 2018-07-30

**Authors:** Primo Proietti, Massimo Trabalza Marinucci, Alberto Marco Del Pino, Roberto D’Amato, Luca Regni, Gabriele Acuti, Elisabetta Chiaradia, Carlo Alberto Palmerini

**Affiliations:** 1 University of Study of Perugia, Department of Agricultural, Food and Environmental Sciences, Perugia, Italy; 2 University of Study of Perugia, Department of Veterinary Medicine, Perugia, Italy; University of Cincinnati College of Medicine, UNITED STATES

## Abstract

Selenium (Se) is an essential element in human and animal diets, based upon a widespread range of beneficial effects that are primarily due to its antioxidant properties. While Se can be associated to anti-cancer and anti-diabetic activities, reproductive efficiency, and enhancement of the immune system, the mechanistic details of the corresponding biological processes are still largely elusive. To avoid deficiencies and increase bioavailability, Se it is generally supplied to livestock through Se-supplemented feeds or forage plants fertilized with inorganic Se. While the relationship between Ca^2+^ and ROS (reactive oxygen species) is well known, only a few studies have addressed the possible involvement of Se in the control of cytosolic Ca^2+^ in oxidative stress. The results on Ca^2+^ homeostasis were obtained adding exogenous Se in the form of SeO_4_^2-^ to sheep lymphomonocytes cultured *in vitro*. In particular, Se strongly attenuated 1mM H_2_O_2_-induced alteration of intracellular [Ca^2+^]_C_ as well as the entry of extracellular Ca^2+^ into the cells with comparable EC50 values for sodium selenate accounting to 1.72 and 2.28 mM, respectively. In an *ex vivo* trial, it was observed that Ca^2+^ homeostasis can effectively be rescued in sheep lymphomonocytes exposed *in vivo* to a Se concentration of approximately 1.9 mM, that was achieved by feeding sheep with olive leaves previously sprayed with 500 mg/plant Na-selenate. Thus the results obtained suggest that the mode of action of selenium markedly influenced Ca^2+^-related signaling events. Furthermore, results clearly reveal that the protective effect of Se on Ca^2+^ homeostasis under oxidative challenge can be clearly and effectively achieved through an appropriate dietary regimen obtained also in a circular economy logic using pruning of olive trees treated to reduce tree drought stress.

## Introduction

Selenium (Se) is a trace chemical used as a dietary supplement in humans and animals. Initially regarded as a toxic molecule, it was subsequently shown to play a role in a number of biological processes across a variety of different experimental models [1, 2]. In this regard, Se has been described as an anticancer and cardio-protective element [3, 4], as well as an anti-diabetic agent based on regulatory implications within crucial events of the glucose metabolism, namely the glycolysis and gluconeogenesis pathways and the insulin response [5].

In ruminants Se deficiency has been mainly associated with nutritional muscular dystrophy (“white muscle disease”) (BIB) [6]. It was also shown to have beneficial effects towards reproductive efficiency in terms of placental size and time of delivery, incidence of metritis and ovarian cysts [7, 8], in vitro Leydig cell functions [9], prevalence of retained fetal membranes [10, 11] and calf mortality [12].

A number of studies have demonstrated that Se is involved in the physiological response to heat stress [13], immune system function [14, 15, 16] and antioxidant defence system [17, 18, 19]. Indeed, in the form of selenoproteins, Se can also be considered a powerful antioxidant, for protection against oxidative stress due to an excess of reactive oxygen species (ROS) and reactive nitrogen species (RNS) [20]. However, the functional implication of most selenoproteins is still unknown.

It must be noticed that, to date, details of Se-related benefits remain largely elusive, suggesting that an arbitrary and poorly controlled intake from the diet or alternative sources could compromise an expected beneficial effect and increase toxicity risks [21]. Reports have shown that biological implications of Se can be concentration-dependent. Excessive exposure to Se may cause anemia [22] due to Se binding to the β-subunit of haemoglobin and, consequently, alteration of carbamate formation resulting from the reaction between CO_2_ and primary amino groups [22]. Se toxicity has been also reported in animal species dependent on its chemical form and concentration [23, 24].

Indeed, the amount of metabolically active Se may depend on the nature of the chemical entities ingested (organic compounds, such as selenomethionine and dimethylselenide, and inorganic selenites and selenates), which can be subject to differential bioavailability and tissue distribution [25].

In many areas of Europe, and particularly in Germany, Denmark, Scotland and Italy, Se concentration in forages is low due to its scarce presence in the soil [26, 27] which, in turn, is related to a number of factors such as high pH, large amount of oxygen, low organic matter and clay content [26].

To avoid deficiencies, Se is usually provided to livestock through mineral mixes, Se-enriched concentrate feeds, or forage plants fertilized with inorganic Se [27, 28, 29]. The bioavailability of the organic Se (mostly Se-methionine) incorporated into plants is known to be greater than the inorganic Na-selenite and Na-selenate usually included in livestock feeds [30, 31]. In addition, when the diet of dairy ruminants is supplemented with Se, milk Se concentration is improved with potential benefits for the consumers [32].

While Se functionality appears to be most likely related to an antioxidant potential, and despite the well-established interplay between Ca^2+^ and ROS [33], only a few studies have addressed the possible involvement of Se in the control of cytosolic Ca^2+^ [34, 35]. Indeed, ROS were shown to cause a dynamic change of cytosolic Ca^2+^ in various types of cells [36], as a result of ion mobilization from Ca^2+^ stores and the subsequent entry of extracellular Ca^2+^, with consequences on the cellular processes in which the Ca^2+^ is involved (cell proliferation, differentiation, apoptosis, and regulation of signaling pathways) [37, 38, 39].

In this work, to clarify the relationship between Se, oxidative stress and Ca^2+^, the effect of Se on Ca^2+^ homeostasis in sheep lymphomonocytes exposed to hydrogen peroxide was investigated. The study was performed, in vitro, upon treatment of cells with exogenous Se, as well as in fresh lymphomonocytes isolated from sheep previously exposed to a diet that included selenate-treated leaves resulting from pruning of olive trees treated with Se in order to reduce tree drought stress [40, 41, 42].

## Materials and methods

### Reagents

FURA 2-AM (FURA-2-*pentakis* (acetoxymethyl) ester, Triton X-100 (t-octylphenoxypolyethoxyethanol), EGTA (ethylene glycol-*bis* (β-aminoethyl ether)-N,N,N’,N’-tetracetic acid), Trypan blue, and Iscove’s Modified Dulbecco Medium were purchased from Sigma-Aldrich Corporation (St. Louis, Missouri, USA). DMSO (dimethyl sulfoxide) All other reagents were of the highest available grade commercially available.

### Experimental study design

#### Plant material

Olive leaves used in the sheep diet for experimental purposes were from 20 year old trees (cv Leccino), grown in central Italy and annually fertilized with 16.5 t ha^-1^ cow manure containing 0.018 mg kg^-1^ of total Se. Total Se concentration in the soil was 0.010 mg kg^-1^. Trees were sprayed in summer with 5 L/plant solution containing 100 mg L^−1^ Se obtained by dissolving Na-selenate (Sigma-Aldrich Corporation, St. Louis, Missouri, USA, cat. n. S0882-25g) in deionized water [43]. This treatment was enhanced by a wetting agent (Wetting PLUS 0.05%, Dow AgroSciences, Italy). For control purposes, a number of olive trees, grown in the same field and subjected to the same cultivation practices, were sprayed under the same conditions but in the absence of Na-selenate.

#### Determination of total Se in olive leaves, hay and blood

Measurements of total Se content in sheep blood and olive leaves were performed using defrozen and dry samples, respectively. Samples of blood (0.5 g/sample), leaves (0.25 g/sample) and hay (0.25 g/sample) were microwave digested (ETHOS One high-performance microwave digestion system; Milestone Inc., Sorisole, Bergamo, Italy) with 8 mL of ultrapure concentrated nitric acid (65% w/w) and 2 mL of hydrogen peroxide (30% w/w). The heating program for the digestion procedure was 30 min with power of 1000 W and 200°C. After cooling down, the digests were diluted with water up to 20 mL, then passed through 0.45 μm filters. The analysis were conduct using a graphite furnace atomic absorption spectrophotometry, Shimadzu AA-6800 apparatus (GF-AAS; GFA-EX7, Shimadzu Corp., Tokyo, Japan) with deuterium lamp background correction and a matrix modifier (Pd(NO_3_)_2_, 0.5 mol L^−1^ in HNO_3_). All analyses were carried out in triplicate [44].

#### Animals and dietary treatment

The experiment was carried out at the “Azienda Zootecnica Didattica”of the Department of Veterinary Medicine, University of Perugia. Twenty Sarda ewes in mid lactation (3^rd^-4^th^ month after parturition) were randomly divided into two groups of equal size, balanced for body weight (41.3±0.9 kg) and body condition score (2.3±0.1), and fed one of the following isoenergetic and isonitrogenous concentrates: 1) a standard pelleted feed (OLIVE), which contained ground dehydrated untreated olive leaves (204.0 g kg^-1^); 2) as in OLIVE, except that the olive leaves were obtained from sodium selenate-treated (OLIVE-Se) trees (Se content in leaves: 7.83±0.13 mg kg^-1^).

All diets were administered for 60 days. In both groups, concentrate was fed at a rate of 350 g per head per day and was administered in two equal parts during the day. The ewes were adapted to the new feed progressively: during the first 15 days of the trial, the content of olive leaves in the concentrate was limited to 85.0 g kg^-1^. A third group of 10 ewes were subjected to the same dietary treatment, except that the concentrate did not contain olive leaves (CTR).

The concentrate contained 167.8 g kg^-1^ crude protein and 290.9 g kg^-1^ neutral detergent fiber and was entirely consumed by the ewes during the whole length of the experiment. Lucerne hay (crude protein: 143.5 g kg^-1^; neutral detergent fiber: 461.3 g kg^-1^) was provided *ad libitum*. Estimated hay dry matter intake was 1.51, 1.45, and 1.48 kg/ewe/day for the CTR, OLIVE and OLIVE-Se group, respectively, and was not affected by the dietary treatment.

Samples of the feeds were collected at 0, 15, 30, 45 and 60 days of the trial and analysed for chemical composition following AOAC methods [45, 46, 47]. The calcium content was determined according to Julshamn et al. [48].

All ewes from the three dietary groups were blood sampled from the jugular vein before the morning meal at the end of the experiment. Blood samples were immediately transported to the laboratory on ice and processed upon arrival.

#### Ethic rules

The study was conducted in accordance with Legislative Decree No. 146, implementing Directive 98/58/EC of 20 July 1998 concerning the protection of animals kept for farming purposes [49]. The Bioethics Committee of the University of Perugia approved the study protocol.

#### Preparation of sheep lymphomonocytes

Whole blood samples were collected from sheep in heparinized Vacutainers tubes (BD). Peripheral blood lymphomonocytes were isolated by Ficoll^®^ Paque Plus (GE Healthcare Life Sciences) density gradient centrifugation. After centrifugation, the PBMC layer was collected and washed in Dulbecco’s phosphate-buffered saline (DPBS) (Sigma-Aldrich) and than re-suspended (10^6^ cells/mL) in Hank's Balanced Salt Sodium (HBSS) Ca^2+^ free (Sigma-Aldrich).

Sheep blood and lymphomonocytes isolated from animals that were exposed to diets featuring different were so named. C1: a standard pelleted feed without olive leaves (CTR); C2: as in C1, except that with added olive leaves (OLIVE); C3: as in C2, except that with added olive leaves plus Se (OLIVE-Se).

#### Measurement of cytosolic Ca^2+^

Intracellular calcium levels were determined spectrofluorometrically using FURA-2AM the probe [50].

Sheep lymphomonocytes were harvested by centrifugation at 800 g for 10 min and then resuspended in 1 mL Ca^2+^-free HBSS buffer (120 mM NaCl, 5.0 mM KCl, MgCl_2_ 1mM, 5 mM glucose, 25 mM Hepes, pH 7.4). Cell suspensions were incubated in the dark with FURA-2AM (2 μL of a 2 mM solution in DMSO) for 60 min, after which samples were centrifuged at 800 g for 5 min. Cells were then harvested and resuspended in 3 mL of Ca^2+^-free HBSS containing 0.1 mM EGTA, which was included to rule out or, at least, minimize a potential background due to contaminating ions.

The Ca^2+^-FURA complex was excited by a dual-view wavelength splitter at 340nm and 380nm, and fluorescence subsequently detected at 510 nm. The ratio between 340-380nm to 510 nm emission was used to determine cytosolic calcium [Ca^2+^]_c_ concentrations [50]. Based upon a 200s exposure to the detector, changes in cytosolic calcium were expressed as Δ[Ca^2+^]_c_ (nM). In this regard, we employed a “Ca^2+^ add-back” approach to study the effects of H_2_O_2_ on the depletion of intracellular Ca^2+^ stores as well as Ca^2+^ entry into the cells. “Ca^2+^ add-back" implies an experimental procedure conducted in two steps: initially, Ca^2+^ is not included in the incubation medium; subsequently, CaCl_2_ is added to challenge the cellular system in order to obtain information about Ca^2+^ entry in cells [51].

Fluorescence was measured in a Perkin-Elmer LS 50 B spectrofluorometer (ex. 340 and 380 nm, em. 510 nm), set with a 10 nm and a 7.5 nm slit width in the excitation and emission windows, respectively. Fluorometric readings were normally taken after 300–350 s.

In all instances, cell-based assays implied cell viability rates that consistently exceeded 95%, as displayed by the Trypan Blue Exclusion method.

#### Statistics

Results obtained from biological replicates are expressed as mean values ± standard error of the mean (SEM). Statistical significance was determined by two-way ANOVA with Tukey’s post-hoc test for multiple comparisons. Statistical tests were performed using Graph Pad Prism 6.03 software for Windows (La Jolla, CA).

## Results

Levels of cytosolic calcium [Ca^2+^]_c_ in sheep lymphomonocytes subject to oxidative stress by hydrogen peroxide.

**Fig 1:**
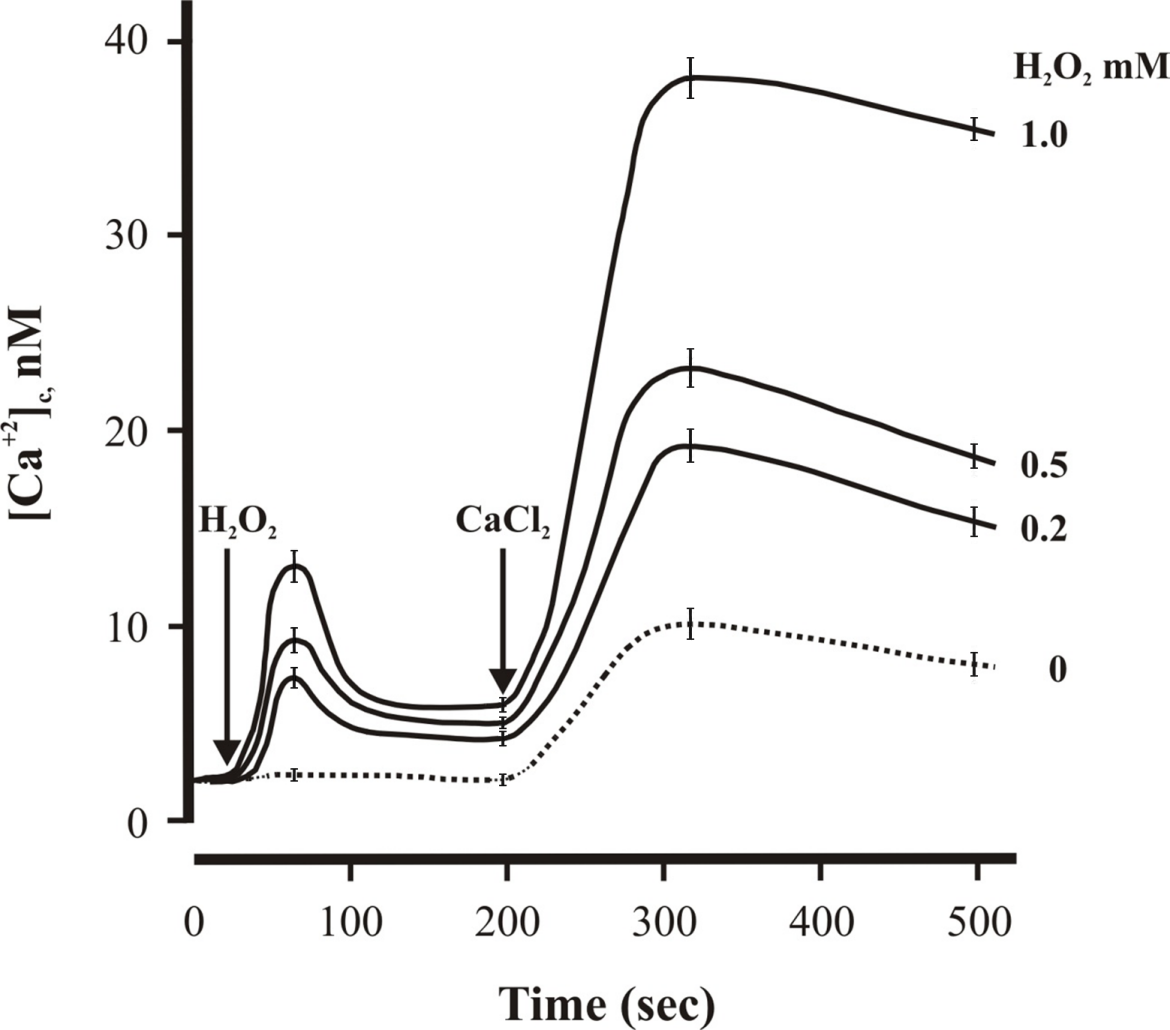
Oxidative stress by H_2_O_2_ in sheep lymphomonocytes. Time-course of sheep lymphomonocytes (1x10^6^) after treatment with H_2_O_2_ (0.2, 0.5,1.0 mM) and, 200s later, 1mM CaCl_2_. Shown are levels (nM) of cytosolic calcium, measured at different time points. Each value calculated and expressed as the means from 5 experiments (biological replicates) ± SEM.

Basal [Ca^2+^]_c_ levels in sheep lymphomonocytes were in the 4–5 nM range however, in the presence of hydrogen peroxide (H_2_O_2_), we observed a rapid(100s) spike in [Ca^2+^]_c_ followed by a quick return to baseline values. Sharp rises in calcium levels were also found when the tissue culture medium was supplemented with increasing doses of H_2_O_2_, as shown in Fig 1.

Both in the absence and presence of added Ca^2+^, the increase in [Ca^2+^]_c_ was found to correlate with the amount of H_2_O_2_ used to challenge the cells (Fig 1), thereby suggesting that, in sheep lymphomonocytes, the oxidative stress caused by H_2_O_2_ had an impact on Ca^2+^- stores depletion and deregulation of Ca^2+^-entry.

Assessment of [Ca^2+^]_c_ in sheep lymphomonocytes exposed to with selenium during H_2_O_2_-mediated oxidative stress.

Above, we showed that H_2_O_2_-induced oxidative stress affects the levels of cytosolic Ca^2+^ and Ca^2+^- entry in sheep lymphomonocytes. Since Se must be considered an essential constituent of the sheep diet [52], we decided to test whether this trace element would specifically be involved in the alteration of Ca^2+^ homeostasis.

Sheep lymphomonocytes labelled with FURA-2 were washed, transferred to Ca^2+^-free medium, and then treated with variable doses (0.01–10 μM) of SeO_4_^2-^. Under these conditions, [Ca^2+^]_c_ was not subject to changes.

**Fig 2:**
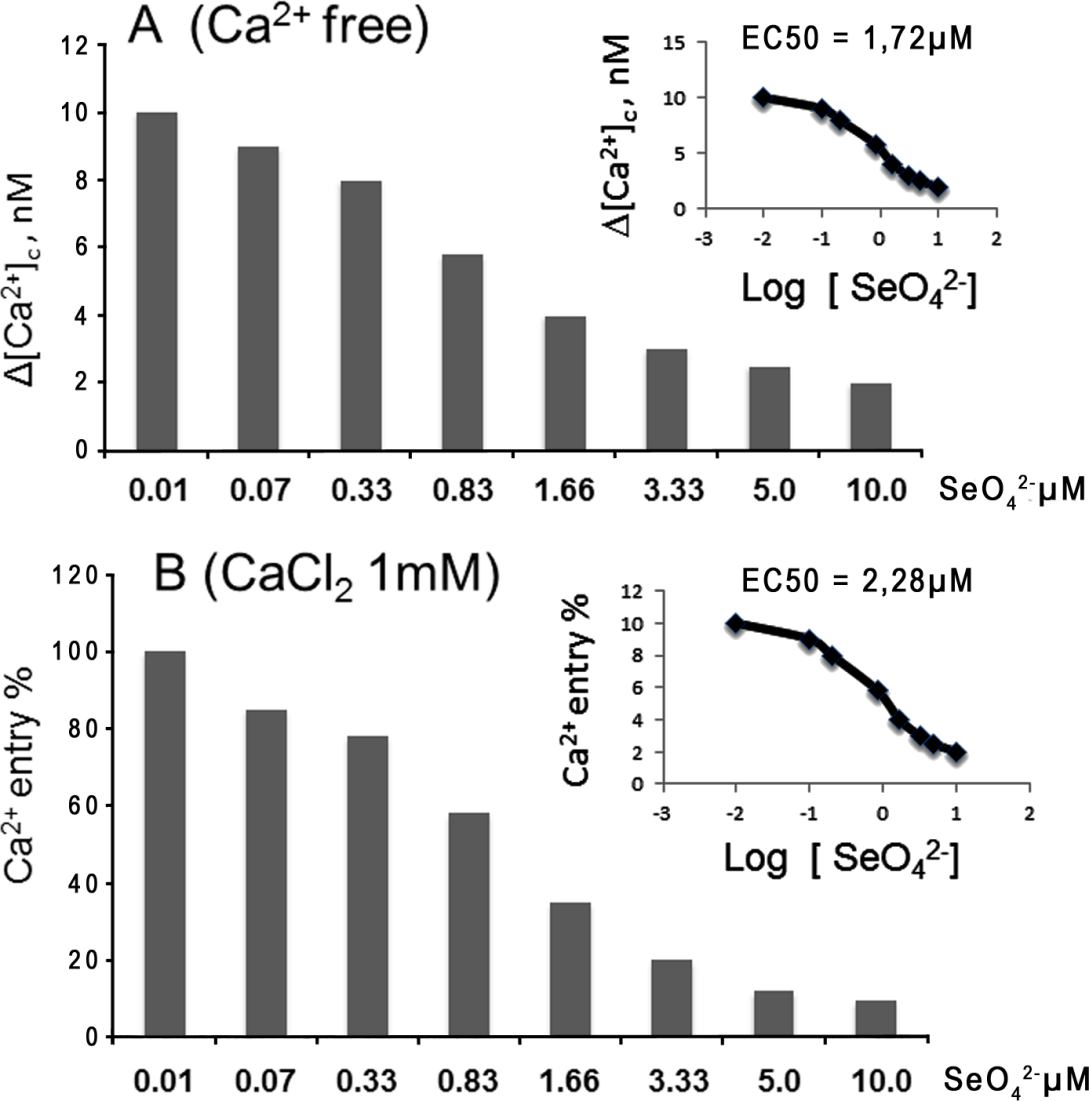
SeO_4_^2-^ effect on calcium homeostasis during H_2_O_2_-induced oxidative stress. Increasing concentrations (0.01–10μM) of SeO_4_^2-^ were employed to pre-treat sheep lymphomonocytes prior to inducing oxidative stress with 1mM H_2_O_2_. Panel A shows the impact of selenium on [Ca^2+^]_c_, and Panel B on Ca^2+^-entry. In both instances, results are expressed in relation to samples that were not exposed to SeO_4_^2-^. Each value corresponds to the mean calculated from 5 biological replicates ± SEM.

However, when cells, pre-exposed to 0.01–10 μM SeO_4_^2-^, were subsequently challenged with 1.0 mM H_2_O_2_, the impact of oxidative stress appeared markedly reduced in relation to [Ca^2+^]_c_ and, more emphatically, Ca^2+^ entry (Fig 2A and 2B). The effect of Se was dose-dependent, with EC_50_ of 1.72 and 2.28 μM, respectively.

These results strongly suggest that selenium counteracts the effect of 1 mM H_2_O_2_ upon the depletion of Ca^2+^ stores and the resulting release of the ion into the cytosol. Similarly, it is shown that selenium can significantly offset H_2_O_2_-mediated Ca^2+^-entry into the cell.

Cytosolic calcium during H_2_O_2_-mediated oxidative stress in sheep lymphomonocytes exposed to Se *in vivo*.

Next, to better assess the nutritional role of Se in calcium homeostasis, we investigated sheep lymphomonocytes isolated from animals that were exposed to diets featuring different levels of the trace mineral.

Table 1 shows the levels of total selenium in olive leaves and concentrate. Clearly, the content of selenium in the leaves(Se-enriched) and pelleted food obtained from sodium selenate-treated trees (OLIVE-SE) was significantly higher than controls.

**Table 1:** Total Se contained in feeds.

Total Se (mg kg ^-1^)				
Olive leaves		Concentrate		Hay
Control	Se-enriched	OLIVE	OLIVE-Se	
0.46±0.098	7.83±0.128	0.12±0.018	0.69±0.012	0.24 ±0.050

OLIVE: pelleted feed containing ground dehydrated olive leaves

OLIVE-Se: pelleted feed containing ground dehydrated olive leaves obtained from sodium selenate-fertilized trees

**Fig 3:**
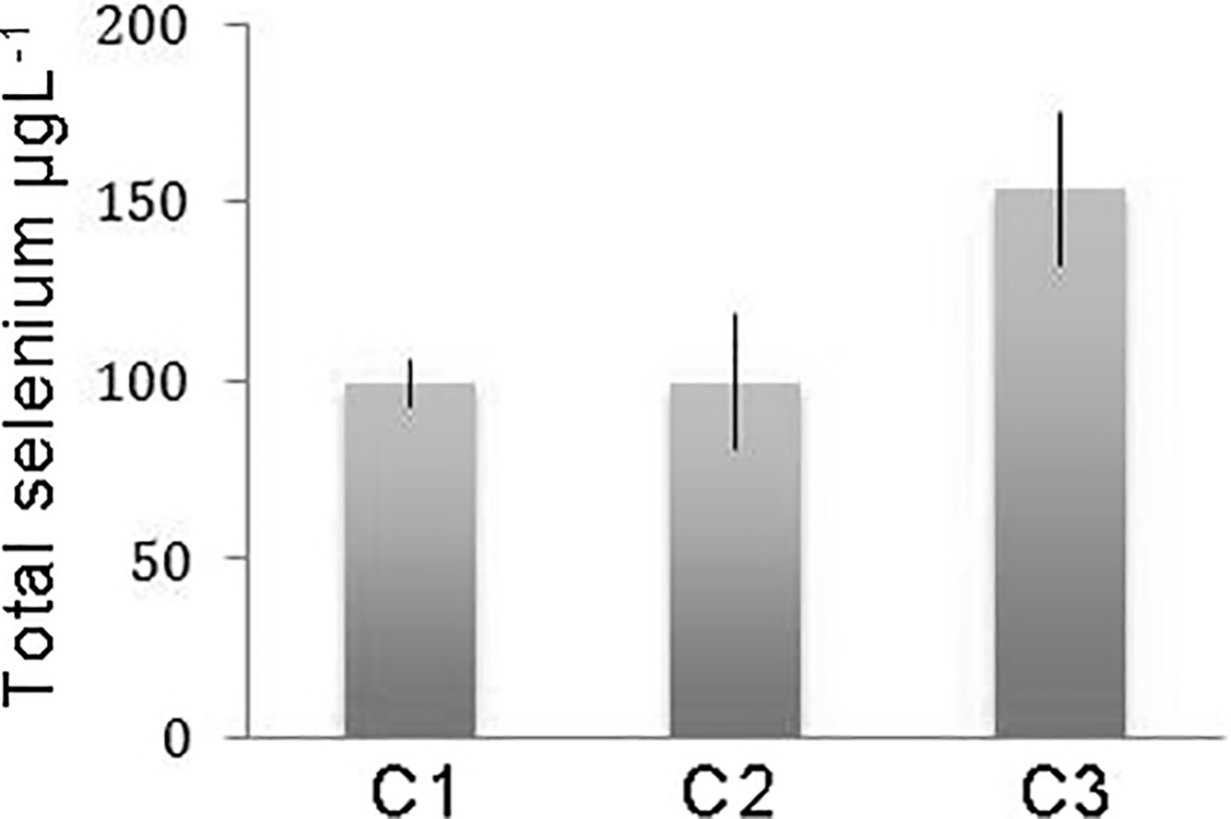
Total Se content in sheep blood. Tests for total Se content were performed in the blood of sheep that were exposed to diets featuring different: C1, C2, C3. Each value corresponds to the mean calculated from 5 biological replicates ± SEM.

As shown in Fig 3, higher levels of Se occurred in the C3, as compared to the C1 and C2 controls. Based on Se molecular mass of 78.96 Da, Se concentration in C3 blood accounted to approximately 1.9 μM.

**Fig 4:**
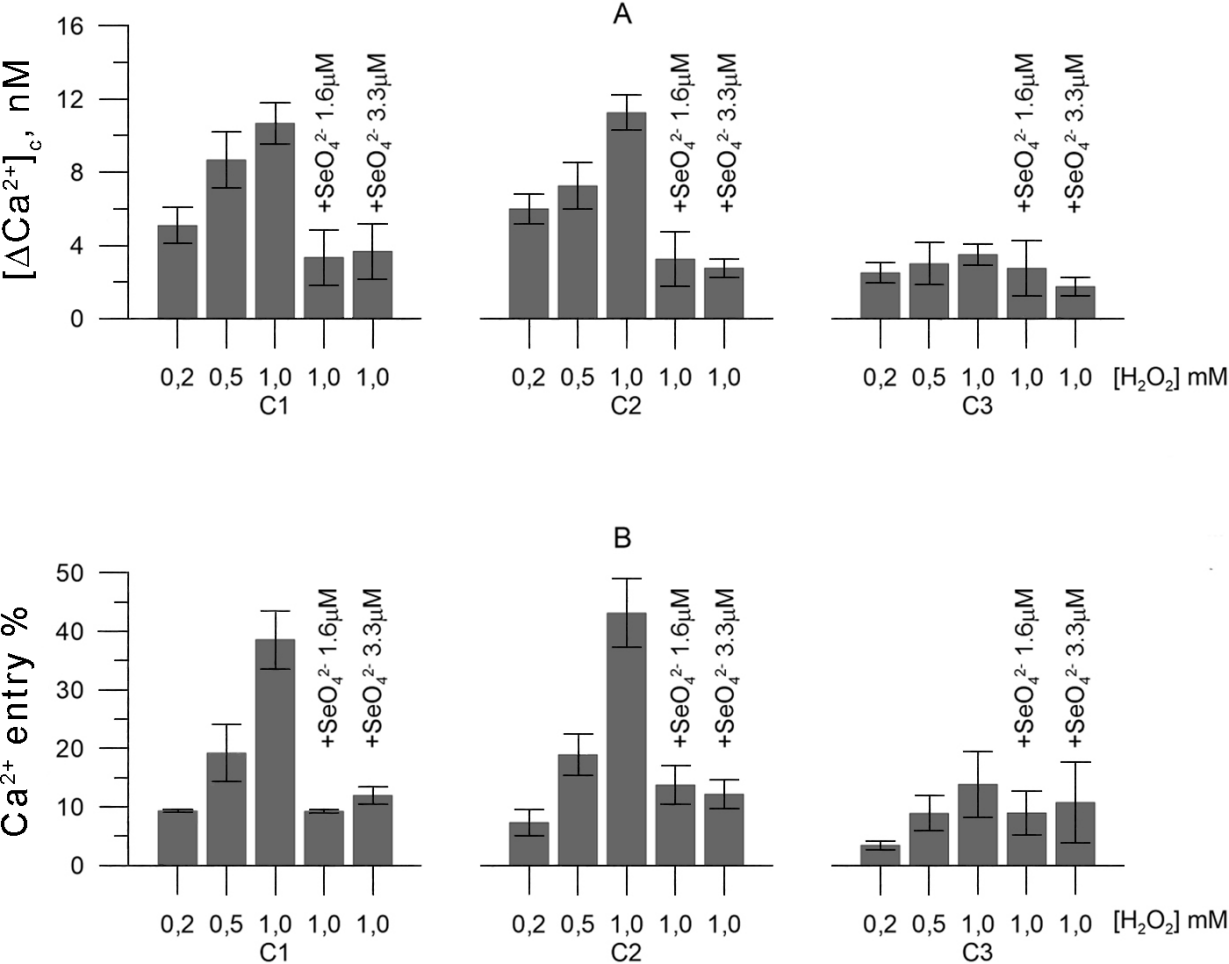
Relevance of dietary Se intake to calcium homeostasis in sheep lymphomonocytes subject to H_2_O_2_-induced oxidative stress. Panel A (Ca^2+^ free): variations of Ca^2+^ cytosolic, and Panel B on Ca^2+^-entry.Each point corresponds to the average of five biological replicates (± SEM) measured on five preparations of lymphocytes obtained from the blood of five different animals included in the project.

Within this context, C1 and C2 lymphomonocytes treated with different amounts of H_2_O_2_ (0.2, 0.5, 1mM), clearly displayed levels of cytosolic calcium that increased with increasing doses of H_2_O_2_. However, this trend was undoubtedly counteracted by 1.6 or 3.3 μM SeO_4_^2-^ dispensed in the tissue culture medium. On the other hand, under the same experimental conditions, C3 lymphomonocytes previously exposed *in vivo* to approximately 1.9 μM Se (Fig 3) showed markedly reduced [Ca^2+^]_c_ that compared well with the results obtained in the presence of either 1.6 or 3.3 μM SeO_4_^2-^ (Fig 4A). These observations paralleled those obtained by measuring Ca^2+^ entry into the cells (Fig 4B).

Taken together, the results unequivocally suggest that Se introduced in sheep diets can largely counteract the effects of oxidative stress on Ca^2+^ homeostasis.

## Discussion

The reactive nature of reactive oxygen species (ROS) can cause irreparable damage to cellular components and eventually disrupt the integrity of the cell membrane. As an oxidant, hydrogen peroxide can induce both apoptosis and necrosis of cells as a consequence of aberrant Ca^2+^ homeostasis associated with the release of Ca^2+^ from intracellular Ca^2+^ stores and/or an abnormal entry of the ion from the extracellular space [36].

In this work, we employed hydrogen peroxide to experimentally induce oxidative stress in sheep lymphomonocytes and, consequently, assess whether marked alterations of Ca^2+^ homeostasis could be rescued by Se, which was either added to the lymphomonocytes as an exogenous reagent, SeO_4_^2-^, or appropriately included in the animals’ diet in order to physiologically impact lymphomonocytes.

To better discriminate the implication of Ca^2+^- stores depletion in relation to Ca^2+^ entry from the outer space, we employed a "Ca^2+^ add-back" protocol based on the supplementation of 1mM CaCl_2_ in the tissue culture medium in order to enhance the detection of mechanisms related to an inflow of exogenous ion or, alternatively, a store-operated release of intracellular Ca^2+^ [51].

The results indicate that, in sheep lymphomonocytes, H_2_O_2_ elicit a dose-dependent increase of cytosolic Ca^2+^ under Ca^2+^ free conditions as well as a result of CaCl_2_ supplementation in the incubation medium, thereby suggesting that H_2_O_2_ can effect both Ca^2+^ mobilization from intracellular stores as well as its entry through the cell membrane. These observations essentially recapitulate what others had reported in different cell types [51, 53]. However, here we found that Se can clearly antagonize either effect of H_2_O_2_ *in vitro* with comparable EC50 values (Fig 2), suggesting a direct impact of Se on Ca^2+^ homeostasis. Furthermore, we observed that Ca^2+^ homeostasis could effectively be rescued by Se introduced as part of the animal’s diet, which we experimentally designed by treating olive leaves with sodium selenate as the only source of that particular trace element. Notably, a concentration of Se in blood sheep accounting to 1.9 μM impacted Ca^2+^ homeostasis similarly to 1.6–3.3 μM SeO_4_^2-^ added exogenously to the incubation medium. Furthermore, we observed that two control groups (C1 and C2), exhibited identical patterns of altered, H_2_O_2_-induced, Ca^2+^ homeostasis, thereby suggesting that any protecting activity shown by Se-treated leaves did not imply any contribution from other anti-oxidants contained in food such as, for example, natural phenols and polyphenols.

Thus, these observations point to a protecting impact of Se against deregulation of Ca^2+^ homeostasis prompted oxidative stress.

Considering that treatment with Na-selenate is used to reduce the stress caused by drought in the olive tree [40, 41], in a circular economy logic, the leaves could been used as a Se vector in sheep feeding with the aim of providing an organic source of Se with greater bioavailability [30, 31] and at the same time contributing to the disposal of pruning by-products [54, 55].

In conclusion, we show that the dietary intake of Se can largely offset the effects of oxidative stress on Ca^2+^ homeostasis, thus elucidating, mechanistically, its beneficial role as an anti-oxidant. Thus, given the vital role of Ca^2+^ homeostasis in a plethora of physiological events [1, 2], and the likely occurrence of oxidative stress during sheep pregnancy [56] and early lactation [57], this paper further supports the notion that Se is a key element that should routinely be included in a healthy diet also using Se-treated tree by-products.
